# Beyond Traditional RAFT: Alternative Activation of Thiocarbonylthio Compounds for Controlled Polymerization

**DOI:** 10.1002/advs.201500394

**Published:** 2016-05-17

**Authors:** Thomas G. McKenzie, Qiang Fu, Mineto Uchiyama, Kotaro Satoh, Jiangtao Xu, Cyrille Boyer, Masami Kamigaito, Greg G. Qiao

**Affiliations:** ^1^ Polymer Science GroupDepartment of Chemical and Biomolecular EngineeringThe University of MelbourneParkvilleVIC3010Australia; ^2^ Center for Advanced Macromolecular Design (CAMD) and Australian Center for NanoMedicine (ACN)School of Chemical Engineering, UNSWSydneyNSW2052Australia; ^3^ Department of Applied ChemistryGraduate School of EngineeringNagoya UniversityFuro‐cho, Chikusa‐kuNagoya464–8603Japan; ^4^ Precursory Research for Embryonic Science and TechnologyJapan Science and Technology Agency4‐1‐8 HonchoKawaguchi, Saitama332‐0012Japan

**Keywords:** controlled/living polymerization, photochemistry, polymer structures, reversible addition‐fragmentation chain transfer (RAFT), thiocarbonyl chemistry

## Abstract

Recent developments in polymerization reactions utilizing thiocarbonylthio compounds have highlighted the surprising versatility of these unique molecules. The increasing popularity of reversible addition–fragmentation chain transfer (RAFT) radical polymerization as a means of producing well‐defined, ‘controlled’ synthetic polymers is largely due to its simplicity of implementation and the availability of a wide range of compatible reagents. However, novel modes of thiocarbonylthio activation can expand the technique beyond the traditional system (i.e., employing a free radical initiator) pushing the applicability and use of thiocarbonylthio compounds even further than previously assumed. The primary advances seen in recent years are a revival in the direct photoactivation of thiocarbonylthio compounds, their activation via photoredox catalysis, and their use in cationic polymerizations. These synthetic approaches and their implications for the synthesis of controlled polymers represent a significant advance in polymer science, with potentially unforeseen benefits and possibilities for further developments still ahead. This Research News aims to highlight key works in this area while also clarifying the differences and similarities of each system.

## 1 Introduction

The use of organic sulphur compounds for the synthesis of synthetic polymers via “living” radical polymerization was pioneered by Otsu and co‐workers over 30 years ago.^1^ Many of the ideas and concepts proposed by Otsu have greatly influenced the development of radical polymerization techniques in what would subsequently emerge as the field of ‘reversible deactivation radical polymerization’ (RDRP).^2^ For RDRP reactions to be successful in producing polymers of uniform molecular weights and high chemical fidelity, a few key criteria must be met. Firstly, the lifetime of the active radical species must be minimized, which can be achieved either via a dynamic equilibrium of reversible (de)activation, or by a process of degenerative chain transfer. Secondly, the rate of initiation must be much faster than that of propagation. The former acts to reduce the likelihood of undesirable termination and/or side reactions occurring, while the latter ensures that all chains grow at an equal rate, decreasing the dispersity of the resulting polymers.^3^ As early as 1984, Otsu observed that if the activation, monomer addition, and recombination (or termination) cycle is carefully controlled, “living” polymerization through a radical mechanism can be approximated.^4^

**Figure 1:**
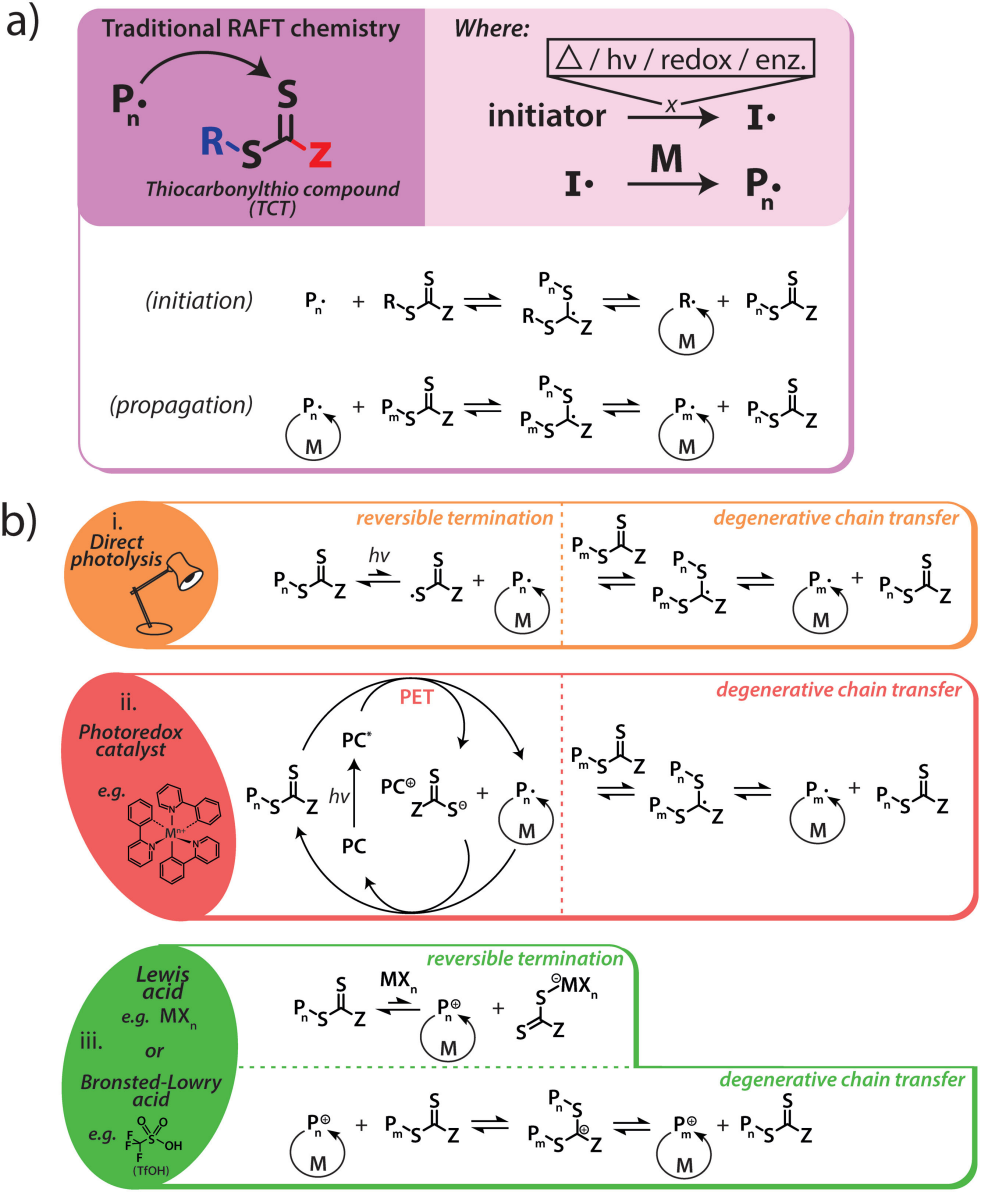
Methods for living polymerization via the activation of thiocarbonylthio (TCT) compounds: a) traditional RAFT polymerization initiated via radical addition. Free radical initiator can be formed by a number of different stimuli; b) recently developed techniques for the activation of TCT compounds for living polymerization reactions: i) direct photoactivation of TCT to initiate a radical polymerization via C–S bond fragmentation, ii) activation via photoredox catalysis resulting in single electron reduction of TCT prior to radical polymerization, and iii) reversible activation via coordination of a Lewis acid for cationic polymerization or activation by Bronsted–Lowry acid to form a cation capable of propagating and undergoing degenerative chain transfer with a suitable TCT compound (note: the initiation step for all polymerizations has been omitted for brevity).

In 1998, Rizzardo and co‐workers reported on the use of thiocarbonylthio (TCT) compounds for the controlled synthesis of polymers via initiation by a conventional radical initiator.^5^ The mechanism of reversible addition‐fragmentation chain transfer (RAFT) was proposed (**Figure** **1**), which can effectively allow for control over the active radical species. Given its relative simplicity with regards to reaction set‐up, its similarity to conventional free radical polymerization, and the excellent structural control observed (i.e., predetermined molecular weight polymers of low dispersity), RAFT polymerization quickly found tremendous uptake by synthetic polymer chemists and has evolved into one of the most widely used RDRP techniques today.^6^ Key to RAFT polymerization is the appropriate selection of a TCT compound (or ‘RAFT agent’). Depending on the structure of the stabilizing (Z‐group) and fragmenting (R‐group) components of the RAFT agent (see Figure 1a) the rates of addition, fragmentation, and subsequent propagation steps will be altered, which ultimately affects and determines the degree of control exerted over the polymerization process.^7^ However, a wide range of RAFT agents have been examined, tested in the literature, and successfully commercialized, allowing access to well‐defined polymers derived from an exhaustive set of monomers with various properties and functionalities, providing the framework for the practical and extensive use of the RAFT technique.

Recent developments in alternative pathways toward TCT activation for use in polymerization reactions have provided significant insight into the roles of each species and the requirement for reaction optimization when multiple competitive reaction pathways are available. In a manner remarkably similar to the original investigations by Otsu, direct photoactivation of compounds containing a TCT moiety has been shown to produce polymers of low dispersity and high end‐group fidelity (i.e., high “livingness”), provided that the combination of monomer, TCT compound, and light source (wavelength and intensity) is appropriately selected.^8^ Alternatively, the use of a photoredox catalyst can effectively facilitate electron transfer to a TCT species, with the resulting electronic instability leading to fragmentation, radical generation, and polymerization.^9^ The versatility of these compounds is demonstrated further by the use of TCTs in cationic polymerizations, where reversible activation is achieved by the coordination of a Lewis acid.^10^ Alternatively, cationic initiation can occur in the presence of a Bronsted–Lowry acid in a manner analogous to traditional radical addition.^11^ This versatility is exemplified by examples of switchable cationic/radical systems within a single reaction vessel.^10^, ^11^ These pathways for activation offer an alternative to the radical addition chemistry traditionally employed for RAFT polymerizations, with the biggest difference being that no additional or exogenous initiator is employed (with the exception of Bronsted–Lowry cationic initiation). Therefore, the TCT in these cases acts as both the initiator and the chain transfer agent. In addition, it is also assumed to act as the reversible terminating agent, thereby fulfilling the roles of an *iniferter* (*ini*tiator‐trans*fer*‐*ter*minator), as described by Otsu.^12^ This distinction has several important implications; most obvious is the avoidance of initiation by means of an exogenous radical or cationic source, which can give unwanted α termini (via direct initiation) or ω termini (via termination of the growing chain by the exogenous radical). Well‐defined polymeric microstructures (i.e., controlled monomeric sequence, high end group fidelity, etc.) are becoming increasingly desirable for a range of high‐tech applications, with a significant drive to develop new polymerization strategies that display fine control over these features. The recent developments for TCT activated polymerizations meet many of these demands. A brief history of each approach will be given below, with the most recent and key works highlighted and discussed in terms of their potential and mechanistic implications.

## 2 Direct Photoactivation of TCTs

The photoactivity of thio‐containing compounds is well known,^13^ and their use by Otsu as photoiniferters paved the way for their subsequent use in polymerization reactions.^1^ Shortly after the first description of RAFT polymerization, initiation of RAFT reactions via irradiation was investigated. For example, reaction mixtures subjected to γ‐radiation can result in a RAFT process due to the formation of radicals derived from monomer, RAFT agent, or solvent.^14^ However, this non‐specific radical generation can lead to various polymer chain imperfections (e.g., branching, different α‐termini, etc.).^15^ In this regard the use of γ‐radiation more closely resembles a traditional RAFT process and therefore differs markedly from the main subject of this highlight article, namely the cases in which the TCT acts as the *sole* source of initiation, in addition to being a key mediating species.

Direct photoactivation of TCT compounds using UV irradiation has also been the subject of much investigation^16^ where ß‐fragmentation of the weakest C–S bond can result in an active carbon‐centered radical and a less active thiyl radical (Figure 1b(i)). However, relatively poor control over the polymerization was commonly observed, especially at moderate to high monomer conversions. This was ascribed to the direct photolytic degradation of the fragmented species (i.e., the thiyl radical), resulting in inefficient deactivation and loss of the “living” character.^17^ These early investigations introduced a debate over the mechanism for the observed “living” behavior, with the two proposed pathways being reversible termination,[[qv: 16b,18]] and the radical initiation of a RAFT‐type degenerative chain transfer process following photolysis of the TCT species.[[qv: 16a]] Key experiments undertaken to elucidate the mechanistic pathway indicate that degenerative chain transfer (i.e., a RAFT process) is likely the main cause of control.[[qv: 16a]] The extent to which reversible termination takes place remains relatively unclear. However, both pathways are typically represented (see Figure 1b(i)), with the predominant mechanism potentially depended on the exact nature of the system. It is pointed out that although the use of exogenous radical photo‐initiators has been demonstrated extensively for the successful photo‐initiation of various RAFT polymerization processes^17^, ^19^ these are not discussed here as this manuscript is focused instead on the *direct* photoactivation of TCT species and the implications thereof.

In 2013, Bai et al. demonstrated that S‐1‐Dodecyl‐S′‐(α, α′‐dimethyl‐α′′‐acetic acid) trithiocarbonate (DDMAT) was capable of controlling the polymerization of methyl acrylate (MA) under UV irradiation, producing narrowly dispersed linear poly(MA) (*Ð* < 1.2) at relatively high monomer conversions.[[qv: 8b]] This report clearly demonstrated that the direct photoactivation of TCTs with UV irradiation could provide a truly robust photopolymerization pathway, and re‐invigorated investigations in this area. In this work, the rate of polymerization was found to be strongly dependent on both the targeted degree of polymerization (DP_n_) (i.e., the initial molar ratio of monomer to trithiocarbonate ([MA]_0_/[TCT]_0_)), as well as the intensity of irradiation. It was also reported that irradiation of a low concentration of TCT (0.45 mm) in the absence of monomer can lead to irreversible degradation of this species, while higher TCT concentrations (13.5 mm) show no structural change even after extended irradiation.[[qv: 8b]] Importantly, fragmentation of the C–S bond in the latter case was still occurring as evidenced by radical trapping experiments, illustrating that the rate of reversible termination was sufficient so as to avoid an extended lifetime of the potentially unstable thiyl radical.^20^ Later that year, Johnson et al. reported a UV‐activated polymerization of *N*‐isopropylacrylamide in the presence of a *bis*‐functional TCT.[[qv: 8a]] The authors demonstrated the “on–off” nature of the photopolymerization, and showed the reaction can be effectively activated under solar radiation – a feature that may be promising for industrial applications where natural light sources are desired or beneficial. Subsequently, Kwark et al. described the UV‐activation of a xanthate (S‐2‐cyano‐2‐propyl‐O‐ethyl xanthate) for the controlled photopolymerization of vinyl acetate (VA).[[qv: 8b]] This system was shown to generate poly(VA) with good control (*Ð* ≈ 1.2–1.3). The rate of the reaction was found to be inversely proportional to the targeted DP*_n_* (with [VA]_0_/[xanthate]_0_ values of 167, 400, and 800 demonstrated), which conflicts with the previous report by Bai et al..[[qv: 8b]]

TCTs are typically strongly yellow, red, or orange in color, which has been ascribed to the *n*→π* electronic transition of the thiocarbonyl double bond, for which the associated absorption often occurs in the visible wavelength region (ca. 400–550 nm).[[qv: 13b]] Recently, the group of Qiao has exploited this phenomenon by selectively exciting the *n*→π* absorption band with blue light (*λ*_max_ ≈ 460 nm), allowing for the visible light photoactivation of a range of TCTs.[[qv: 8f]] Despite the weak molar absorptivity in this region the TCTs investigated were shown to polymerize a range of acrylates and acrylamides, resulting in well‐defined linear polymers (*Ð* < 1.1), with high end group fidelities even at near complete monomer conversions (conv. > 95%) as evidenced by matrix‐assisted laser desorption/ionization‐time of flight mass spectrometry (MALDI‐ToF MS) analysis and in situ chain extension experiments. The Boyer group independently investigated the direct photopolymerization of methacrylates under low intensity (≈0.7 mW cm^–2^) blue and green light irradiation while developing their photoredox catalyzed system (vide infra).[[qv: 8e]] The proposed mechanism for these two techniques was carefully compared. The polymerization kinetics for direct photoactivation is dependent on light wavelengths, intensities, monomers and specific TCT compounds. Visible light activation had been reported by the group of Kamigaito previously,[[qv: 8c]] where irradiation with a weak white light source (a household fluorescent bulb) was demonstrated to activate the polymerization of vinyl monomers in the presence of a xanthate compound (*O*‐ethyl‐*S*‐(1‐ethoxycarbonyl)ethyldithiocarbonate). The authors astutely observed that this may make the employed TCT suitable as a ‘photoiniferter’, however further investigation was not pursued. However, a similar system was recently reported in which various xanthates were used for the blue light mediated photopolymerization of the unconjugated monomer vinyl acetate.^21^ Excellent control over the polymerization was observed with a high degree of chain end fidelity as determined by MALDI‐ToF analysis.

Although there exist some benefits to using visible light rather than UV as the source of activation (e.g., avoidance of monomer self‐initiation, experimental safety, etc.), the potential mechanistic and structural benefits (i.e., minimization of side/termination reactions or direct thiocarbonyl degradation) require further examination. Regardless, this approach employing solely TCT compounds as the photoactive species and control agent offers the potential for further modification, optimization, as well as a deeper understanding of the competing reaction pathways.

## 3 TCT Activation via Photoredox Catalysis

**Figure 2:**
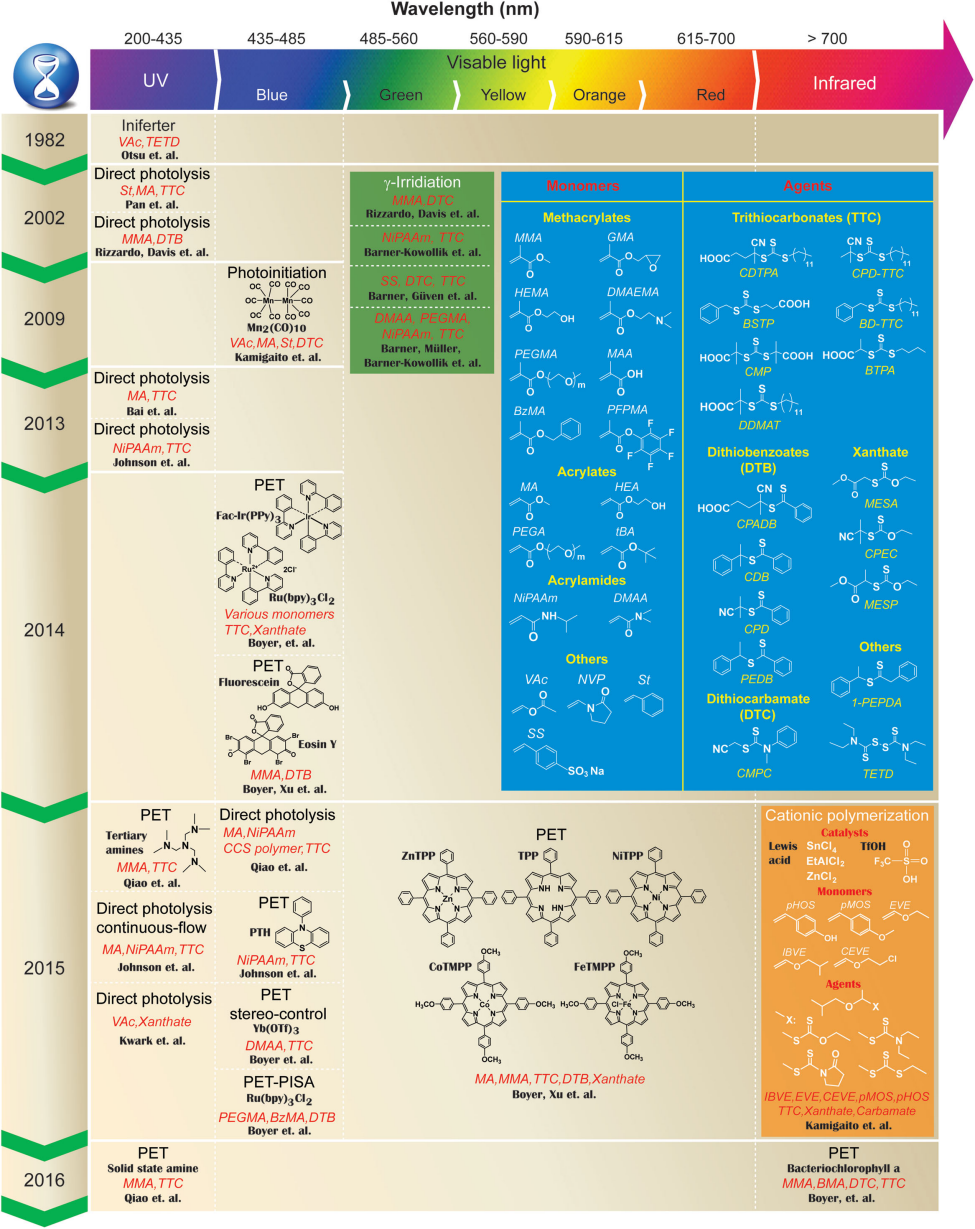
Chronology of various monomers, TCT compounds, activators, and light sources investigated for photocontrolled radical polymerizations. Acid activators, monomers, and TCTs investigated for cationic polymerization are shown in bottom right.

The Boyer group recently introduced the concept of TCT activation via a photoinduced electron transfer (PET) process by incorporating a suitable photoredox catalyst with/without an electron donor.[[qv: 9a]] The employed photoredox catalyst is proposed to reduce the TCT to generate a radical anion, along with the catalyst in an elevated oxidation state. The resulting TCT anion can fragment to give an initiating carbon‐centered radical, before back electron transfer (re)generates the dormant polymer chain and the photocatalyst in its ground state, thereby completing the catalytic cycle (see Figure 1b(ii)). The term PET‐RAFT was coined for these types of photopolymerization systems, which have subsequently been demonstrated to be widely applicable to a variety of catalysts and monomer families (**Figure** **2**). In some cases, after excitation the photoredox catalyst can transfer its energy directly to the TCT to activate it.^22^

Initial investigations into the photoredox activation of TCTs was performed using an iridium complex (*fac*‐[Ir(ppy)_3_], Ir^(III)^),[[qv: 9a]] which has been extensively employed for a variety of organic transformations.^23^ With appropriate stoichiometry, Boyer and co‐workers demonstrated that these systems were able to furnish well‐defined polymers under irradiation from a blue LED (*λ*_max_ = 435 and 460 nm) for a variety of different monomers (both activated and less activated monomers). The high end group fidelity of the resulting polymers was confirmed by ^1^H NMR analysis, UV–vis spectroscopy, and successive chain extension experiments to form (multi)block copolymers. The PET‐RAFT system was subsequently transferred to aqueous conditions through use of a water‐soluble catalyst (Ru(bpy)_3_Cl_2_, Ru^(II)^).[[qv: 9b]] This allowed for the efficient preparation of protein‐polymer conjugates with a high enzymatic activity.

During their investigations, it was observed that the PET‐RAFT polymerization could be performed even in the presence of molecular oxygen (i.e., without degassing).[[qv: 9a,c,g]] Given that radical polymerizations are typically inhibited by oxygen due to its excellent radical scavenging behavior, the ability to perform controlled radical polymerization without the need for thorough degassing of the reaction mixture would represent a major advancement in the applicability of these systems. The strong photoreducing nature of the employed catalysts is thought to allow for the single electron reduction of dissolved oxygen, consuming any inhibiting oxygen species prior to the polymerization taking place. Photopolymerizations performed in air (i.e., in the presence of oxygen) were demonstrated to occur with minimal difference in reaction rate between deoxygenated and non‐deoxygenated reaction mixtures.[[qv: 9a]] An increased induction period was observed in the non‐deoxygenated mixtures for both acrylate and methacrylate monomer types, which was ascribed to the time during which molecular oxygen was consumed. The length of inhibition period is proportional to the volume of air present in the reaction flask and the concentration of the catalyst.

A potential drawback of the initially reported PET‐RAFT process was the expensive and potentially toxic transition‐metal complex used (albeit in very small quantities). This was subsequently addressed via the use of a range of abundant and non‐toxic photoactive organic compounds,[[qv: 9d,h]] naturally derived metallo‐complexes (including chlorophyll *a* which could be extracted directly from spinach leaves),[[qv: 9f]] and various porphyrin structures, which greatly enhanced the versatility and applicability of these systems.[[qv: 9g]] The Qiao group recently reported the use of organic amines under UV irradiation for the photopolymerization of methacrylates with both homogeneous[[qv: 9e]] and heterogeneous^24^ amine catalyst systems, that were assumed to act via a similar PET mechanism. A range of cheap and commercially available tertiary amines were demonstrated to be efficient for these reactions, with little effect of amine molecular structure on reaction kinetics provided that the concentration of amine centers was kept constant. These organic photoredox catalysts are important not only in lowering the cost of the reaction, but also to avoid issues of toxic metal catalyst residues remaining in the polymeric products.

Given the diversity of photoredox catalysts demonstrated, focus was then placed on expanding the range of irradiating wavelengths able to activate the polymerization. This can have advantages including lower energy consumption and deeper photon penetration through films and/or gels when longer wavelengths are employed. Tunable photocatalysis based on a single molecule was achieved via the use of a zinc‐porphyrin photoredox catalyst, with the rate of the polymerization linked to the absorption profile of the catalyst and thus tunable over a range of visible wavelengths.[[qv: 9g]] Interestingly, the zinc‐porphyrin employed displayed high activity towards trithiocarbonates, while other TCT compounds (e.g., dithiobenzoate, dithiocarbamate, and xanthate) displayed much slower kinetics, inferring selective polymerization activation. This was believed to suggest some form of novel molecular recognition occurring between the metal‐porphyrin and the trithiocarbonate, and may be similar to the interactions between zinc and sulfur sites in biomolecules and enzymes, where specific interactions mediate certain redox processes, DNA recognition events, and transcriptional activation.[[qv: 9g]] Surprisingly, the photopolymerizations can proceed in the presence of air with a minimal induction period (usually attributed to the time during which oxygen is reduced/eliminated). By using a novel biocatalyst (bacteriochlorophyll *a*) Boyer et al. recently demonstrated a PET‐RAFT system that could be regulated by near infrared (NIR) and far‐red light.^25^ This has the advantage of deeper penetration profiles compared with higher energy shorter wavelengths (e.g., UV, blue) previously employed, and continues the trend of continuously seeking lower energy means of activation (see Figure 2).

Further development of the PET‐RAFT system was illustrated by the synthesis of polymers with varying levels of stereocontrol.^26^ The synthesis of stereoregular polymers via radical polymerization has been a longstanding challenge for polymer chemists, and there exists a strong desire to develop control over polymer tacticity using the simplicity that radical polymerization offers from both an academic and industrial perspective. In this regard, the PET‐RAFT system has been shown to be amenable to such stereocontrol via the simple addition of a Lewis acid mediator (Y(OTf)_3_ or Yb(OTf)_3_), along with an Ir^(III)^ photoredox catalyst. The choice of solvent is crucial in these experiments as the complexation strength of the monomer‐mediator pair determines the extent of stereocontrol. In light of this, Boyer et al. discovered that by gradually adding DMSO—a Lewis base that can interfere with the Lewis acid mediator—the stereocontrol of the polymerization could be modulated in situ, allowing for the preparation of a multiblock copolymer with five blocks of differing stereoregularity.^26^

## 4 Acid Activation for Cationic Polymerization

The Kamigaito group observed that the activation of oxygen‐ester bonds via Lewis acids (LAs) commonly employed for the cationic polymerization of vinyl ethers may be replicable with TCT compounds, allowing for transformations between RAFT radical polymerization and a living cationic polymerization in a single reaction vessel. This hypothesis was confirmed with the reported synthesis of block copolymers consisting of (meth)acrylate and vinyl ether blocks by an in situ transformation from radical initiated RAFT polymerization to a cationic system via the addition of a LA activator.[[qv: 10a]] It was demonstrated that although vinyl ether cannot homopolymerize in a radical system, it can copolymerize with (meth)acrylate units. However, after addition of the LA the vinyl ether is rapidly consumed while no further conversion of the (meth)acrylate units occurs. The observed molecular weights correspond to theoretical values if one TCT compound initiates one polymer chain, indicating that control over these polymerizations is exerted by these species. It is postulated that activation occurs via coordination of the LA to the thiocarbonyl (C = S) moiety, releasing the R‐group as an active carbocation (Figure 1b(iii)). A dynamic equilibrium is therefore established between these species to allow effective control over the chain‐growth polymerization process. It was observed that to be effective in the cationic polymerization the fragmenting group of the TCT compound must be vinyl ether‐derived. This can be accomplished via the synthesis of a unique TCT for this purpose, or alternatively by the copolymerization of a (meth)acrylate with an excess of vinyl ethyl monomer using a traditional RAFT agent, thereby transforming the end group (i.e., the fragmenting species) into a short poly(vinyl ether) segment.

Interconvertible systems with dual cationic and radical propagation mechanisms were subsequently investigated for a series of vinyl ether‐derived TCTs.[[qv: 10b]] These were shown to facilitate the copolymerization of two monomers with vastly different reactivity profiles through a dual activation mechanism, allowing for the synthesis of copolymers that were previously inaccessible. The structure of the TCT Z‐group, as well as the choice of LA and the (co)monomers strongly affects the number of times the mechanism was ‘interconverted’ during a typical chain growth. This directly alters the segment length of each monomer‐type during the copolymerization, imparting different glass transition temperature behaviors.

Using a different approach, Kamigaito et al. also demonstrated the controlled synthesis of poly(vinyl ether) using a strong Bronsted‐Lowry organic acid (triflic acid—CF_3_SO_3_H or TfOH) as an initiator, and a suitable TCT compound as a mediating agent.^11^ The ability of the TCT compound to act via the degenerative addition‐fragmentation of *cationic* species, and to therefore undergo a RAFT mechanism, was demonstrated for the first time (see Figure 1b(iii)), whereas the previous LA‐catalysed systems are assumed to predominantly occur via reversible activation (although some contribution from cationic RAFT cannot be ruled out).^11^ This technique can provide a metal‐free pathway which may be beneficial for applications where metal contamination is undesirable (e.g., bio‐, food or optical applications). A similar approach was reported by Sugihara et al., where an HCl+Et_2_O adduct was employed for the activation of a cationic polymerization.^27^ However, in this study an oxygen‐ester was demonstrated to give the same results as the thio‐ester, suggesting that the mechanism proceeds primarily through a Lewis acid‐type reversible activation rather than a cationic RAFT mechanism.

## 5 Applications

**Figure 3:**
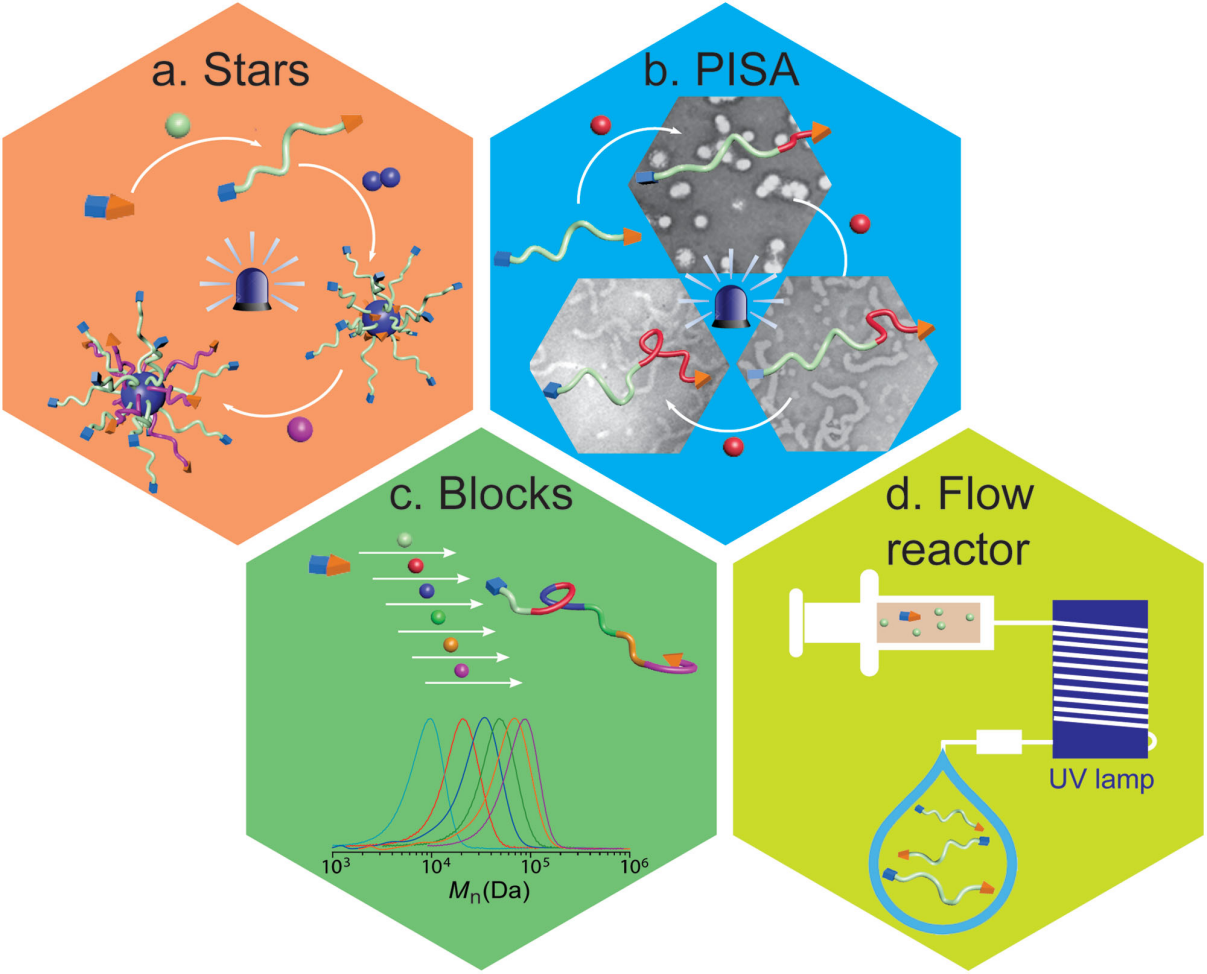
Application of photopolymerization techniques. a) Star polymer nanoparticles via linear macroinitiator growth followed by one‐pot sequential addition of cross‐linker to form stars and further monomer to afford 2nd generation of star arms. b) Polymer nanoparticles of various morphologies formed by polymerization‐induced self‐assembly (PISA) using PET‐RAFT. c) The synthesis of multiblock copolymers via a one‐pot in situ chain extension approach. d) Polymerization in a continuous flow reactor using direct UV photoactivation of thiocarbonylthio compounds.

With each of these new chemistries involving TCT compounds there exists a range of possibilities regarding their expansion in versatility and efficiency. For example, Boyer et al. have already diversified greatly the range of photoredox catalysts that can be employed for the PET‐RAFT system, while one can imagine a similar expansion in the range of (functional) TCTs and monomers in the future. These systems also open new routes to advanced materials and novel structures that may be of interest for a variety of polymer‐based technologies (**Figure** **3**). For example, the Qiao group have demonstrated the use of direct photoactivation of TCTs to synthesize star polymer nanoparticles in a high yielding one‐pot synthesis (Figure 3a).^28^ The excellent chemical fidelity of the resulting polymers was exemplified by the synthesis of miktoarm stars via the “in–out” approach. Kinetic investigations of the TCT photopolymerization indicated a 95% re‐initiation efficiency, thereby ensuring that the second generation of arms were roughly equivalent in number to the first. The Boyer group have also reported the synthesis of polymeric assemblies using the PET‐RAFT system via a polymerization‐induced self‐assembly (PISA) process (Figure 3b).^29^ Multiple morphologies, including spherical micelles, worm‐like micelles, and vesicles, were observed. Importantly, highly pure worm‐like micelles were readily isolated due to the formation of highly viscous gels. More recently, the successful implementation of PET‐RAFT in aqueous miniemulsion afforded a greener approach to prepare polymeric materials.^30^ The high “livingness” of the systems described above also lend themselves well to the synthesis of (multi)block copolymers via an in situ chain extension approach (Figure 3c). This has been demonstrated using radical photopolymerization,[[qv: 8f,9a,26]] and by switching between radical and ionic propagation mechanisms,^10^ allowing for the synthesis of a range of complex and functional multiblock copolymers.Johnson et al. demonstrated the ease with which the photopolymerization of TCTs can be transferred into a continuous flow reactor setup by using direct UV activation (Figure 3d).^31^ They report a four‐fold increase in reaction rate compared with analogous batch reactions due to the change in reactor geometry, with a high surface‐to‐volume ratio allowing for more efficient irradiation, mixing, and heat/mass transfer in the flow system. These benefits apply more generally to all photo‐RDRP techniques,^32^ however the inherent benefit of the homogeneous, catalyst and initiator‐free reaction mixture means that in the system of Jonhson et al. the only species entering the flow reactor are the monomer and the TCT, while the only species exiting the reactor are the polymer product and any unreacted monomer, allowing for simple and scalable collection and purification protocols.

## 6 Conclusions

The versatility of organic sulphur compounds, and in particular TCTs, for chemical transformations and polymerization reactions is rapidly expanding. For polymerizations, the activation of these species through direct photoexcitation, photoredox catalysis, and acid activation have been shown to provide exemplary pathways towards well‐defined polymers with high chemical fidelity provided that the conditions and reagents are selected appropriately so as to optimize the desired reaction pathway. We believe that these TCT‐based techniques offer further unexplored potential for expansion and refinement, and may bring about access to new and high‐value polymer‐based technologies. During the preparation of this manuscript, two comprehensive reviews regarding photopolymerization systems were published by the groups of Johnson^33^ and Yagci^34^, which may be of interest to the reader.
